# A retrospective study comprising 228 cases of pediatric scalp and skull lesions

**DOI:** 10.1186/s12887-023-04231-7

**Published:** 2023-09-20

**Authors:** Lei Yang, Meng-Cheng Yang, Pei-Ran Qu, Di Zhang, Ming Ge, Da-Peng Li

**Affiliations:** 1 Department of Otolaryngology, Head and Neck Surgery, Beijing Children’s Hospital, Capital Medical University, National Center for Children’s Health, Beijing, 100045 China; 2 Department of Urological Surgery, Beijing Children’s Hospital, Capital Medical University, National Center for Children’s Health, Beijing, 100045 China; 3 Department of Neurosurgery, Beijing Children’s Hospital, Capital Medical University, National Center for Children’s Health, No. 56, Nanlishi Road, Xicheng District, Beijing, 100045 China

**Keywords:** Scalp lesion, Skull lesion, Children, Clinical characteristics

## Abstract

**Background:**

Most neurosurgery presentations in children present with a mass that may be scalp and skull lesions, including neoplastic and congenital malformed structural lesions, respectively. Clinicians should make early diagnoses and identify cases requiring surgical intervention promptly to help achieve a better prognosis.

**Method:**

This study retrospectively reviewed studies on children’s scalp and skull lesions within a pediatric medical center’s department of neurosurgery. The detailed clinical information and pathological types of these cases were scrutinized.

**Result:**

A total of 228 children’s scalp and skull lesions with clinical information and identified histopathology types were summarized. The most common scalp and skull lesions were benign dermoid cysts; malignant types were rare but can occur in children.

**Conclusion:**

Based on the combined clinical symptoms and image information, children’s scalp and skull lesions should be diagnosed early. Malignant scalp and skull lesions/other special cases should be treated seriously.

## Background

Most neurosurgery presentations in children present with a mass that may be scalp and/or skull lesions, including neoplastic and congenital malformed structural lesions. Scalp and skull lesions are defined as abnormal extracranial protrusions in the region ranging from the external occipital protuberance to the supraorbital margin; their anatomical characteristics may be superficial or deep and may comprise skin, subcutaneous connective tissue, epicranial aponeurosis, loose areolar connective tissue, the pericranium, and the skull. Neoplasms can form and invade all areas of the scalp and skull anatomy; their pathological nature is always multivarious and may be benign or malignant [1, 2].

Scalp and skull lesions in children may be congenital and present primarily as malformations. In some children, scalp and skull lesions appear following birth; these may be hidden or grow rapidly and can evidence abrupt changes. Some benign scalp and skull lesions tend to grow aggressively, compressing and invading the skull. In the early diagnosis of scalp and skull lesions in children, surgery is the treatment method for most cases. This study focuses on the onset of scalp and skull lesions in children and characterizes the clinical presentations of scalp neoplasms in the pediatric population.

## Materials and methods

The authors retrospectively collected scalp and skull lesion studies involving pediatric patients from 2019 to 2021 in the Department of Neurosurgery in Beijing Children’s Hospital, Capital Medical University, National Center for Children’s Health. All procedures were performed, and written informed consent was obtained in accordance with the relevant guidelines and regulations of the Declaration of Helsinki.

The authors collected detailed information about patients with scalp and skull lesions from electronic medical records with standardized forms comprising basic clinical information, including age at presentation, gender, location and size of the lesions, histopathology and other associated findings, and follow-up information.

Patients often presented to the outpatient clinic for a palpable mass located on the head. Imaging studies confirmed that neoplasms were located within the scalp or skull; the size of neoplasms was ascertained by a combination of palpating, B-scan ultrasonography, and computed tomography (CT) imaging, and the largest value was adopted as the diameter of the neoplasm. All patients underwent surgical lesion treatment under general anesthesia, and the diagnosis of all scalp and skull lesions were determined by histopathological department.

## Results

### Demographic characteristics

The authors summarized cases of scalp and skull lesions treated with surgery during the from 2019 to 2021 in the Department of Neurosurgery, Beijing Children’s Hospital, Capital Medical University, National Center for Children’s Health. A total of 228 cases were included, comprising 127 males and 101 females. The onset position of scalp and skull lesions ranged from the frontal, occipital, temporal, vertex, and fonticulus anterior. The lesion size was randomly distributed; for 71 patients, it was smaller than or equal to 1 cm; for 106 patients, the size ranged from 1 to 3 cm, and for 49 patients, the lesion size was larger than 3 cm. A total of 71 patients presented with symptoms within 1 month of birth, 64 presented with symptoms between the ages of 1 month and 1 year old, 64 patients developed lesions between the age of 1 to 6 years old, and 22 patients developed lesions after the age of 6 years. In terms of patient age on presentation to the clinic, 75 patients were treated before one year old, 118 patients were treated between the age of 1 and 6 years old, and 35 patients were treated between the age of 7 to 12 years old (Table 1).

**Table 1:** Demographic and clinical data of the pediatric patients with scalp and skull lesions

Characteristics	Case numbers
Patients	
Male	127
Female	101
Onset position	
frontal	52
vertex	53
temporal	40
occipital	70
fonticulus anterior	13
Diameter	
less than or equal to 1 cm	71
1-3 cm	106
More than or equal to 3 cm	49
Unknow	2
Age of neoplasm onset	
≤1 month	71
1 month − 1 year	71
1 year – 6 years	64
≥6 years	22
Age of presentation to the clinic	
<1 year	75
1–6 years	118
7–12 years	35

### Histopathology types

Table 2 shows the histopathology types of all 228 patients with scalp and skull lesions. Common lesions included dermoid cysts (108/228 = 44.4%), meningocele (27/228 = 11.8%), Langerhans cell histiocytosis (LCH) (23/228 = 10.1%), myofibroma (12/228 = 5.3%), calcifying epithelioma (9/228 = 3.9%), growth fractures (7/228 = 3.1%), vascular malformations (6/228 = 2.6%), meningioma (4/228 = 1.8%), nevus (4/228 = 1.8%), dermal sinus tracts (4/228 = 1.8%), and neurofibroma (3/228 = 1.3%). Uncommon lesions were found in less than 2% of patients and included Gardner fibroma, hemolymphangioma, hematoma, nodular fasciitis, hemangioendothelioma, neuroblastoma, B-lymphoblastic lymphomas, myeloid sarcoma, hyperosteogeny, granuloma annulare, teratoma, Masson’s pseudoangiosarcoma, neurilemmoma, giant cell fibroblastoma, cholesteatoma, and dermatofibroma.

**Table 2:** Histopathology classification of scalp and skull lesions

Neoplasm	N	Sex	Onset age	Location	Diameter (cm)
Dermoid cyst	108	M:58 F:50	≤ 1 M:35;1 M ~ 1Y:46;1 ~ 6Y:24; >6Y:3	F:21; V:16; T:26; O:34; AF :11	≤ 1:52 ;1 ~ 3:45 ;≥3:11
Meningocele	27	M:18 F:9	≤ 1 M:24;1 M ~ 1Y:2;1 ~ 6Y:1; >6Y:0	F:0; V:9; T:0; O:18; AF :0	≤ 1:6;1 ~ 3: 11;≥3:10
Langerhans cell histiocytosis	23	M:12 F:11	≤ 1 M:0;1 M ~ 1Y:2;1 ~ 6Y:13; >6Y:8	F:7; V:7; T:6; O:3; AF :0	≤ 1:5 ;1 ~ 3:12 ;≥3:6
Myofibroma	12	M:8 F:4	≤ 1 M:0;1 M ~ 1Y:5;1 ~ 6Y:4; >6Y:3	F:5; V:3; T:2; O:1; AF :0; Mu:1	≤ 1:2;1 ~ 3:10 ;≥3:0
Calcifying epithelioma	9	M:4 F:5	≤ 1 M:0;1 M ~ 1Y:3;1 ~ 6Y:5; >6Y:1	F:3; V:4; T:0; O:2; AF :0	≤ 1:3 ;1 ~ 3: 6;≥3:0
Growth fractures	7	M:2 F:5	≤ 1 M:2;1 M ~ 1Y:5;1 ~ 6Y:0; >6Y:0	F:4; V:3; T:0; O:0; AF :0	≤ 1:0 ;1 ~ 3: 1;≥3:4 ; Unknown: 2
Vascular malformation	6	M:5 F:1	≤ 1 M:0;1 M ~ 1Y:1;1 ~ 6Y:4; >6Y:1	F:1; V:2; T:3; O:0; AF :0	≤ 1:1 ;1 ~ 3:3 ;≥3:2
Hemangioma	4	M:2 F:2	≤ 1 M:2;1 M ~ 1Y:0;1 ~ 6Y:2; >6Y:0	F:1; V:1; T:0; O:1; AF :1	≤ 1:0;1 ~ 3: 2;≥3:2
Nevus	4	M:2 F:2	≤ 1 M:2;1 M ~ 1Y:2;1 ~ 6Y:0; >6Y:0	F:0; V:3; T:0; O:1; AF :0	≤ 1:0;1 ~ 3: 3;≥3:1
Dermal sinus tracts	4	M:3 F:1	≤ 1 M:3;1 M ~ 1Y:1;1 ~ 6Y:0; >6Y:0	F:2; V:1; T:0; O:1; AF :0	≤ 1:0 ;1 ~ 3: 2;≥3:2
Neurofibroma	3	M:2 F:1	≤ 1 M:0;1 M ~ 1Y:0;1 ~ 6Y:1; >6Y:2	F:0; V:0; T:0; O:3; AF :0	≤ 1: ;1 ~ 3:1 ;≥3:2
Others	21	M:11 F:10	≤ 1 M:3;1 M ~ 1Y:4;1 ~ 6Y:10; >6Y:4	F:8; V:4; T:3; O:6; AF :0	≤ 1:2 ;1 ~ 3:10 ;≥3:9
Total	**228**	M:127 F:101	≤ 1 M:71;1 M ~ 1Y:71;1 ~ 6Y:64; >6Y:22	F:52; V:53; T:40; O:70; AF :13	≤ 1: 71;1 ~ 3:106 ;≥3:49 ; Unknown: 2

F: frontal; V: vertex; T: temporal; O: occipital; AF: Anterior fontanelle; Mu: multiple

Others: including the uncommon neoplasms of Gardner fibroma, Hemolymphangioma, Haematoma, Nodular fasciitis, Hemangioendothelioma, Neuroblastoma, B-lymphoblastic lymphomas, Myeloid sarcoma, Hyperosteogeny, Granuloma annulare, Teratoma, Masson’s pseudoangiosarcoma, Neurilemmoma, Giant cell fibroblastoma, Cholesteatoma, Dermatofibroma

Overall, 108 cases of dermoid cysts were histologically ascertained; in most patients (81/108 = 75.0%), this occurred below the age of 1 year. The chief complaint in these cases was of an inadvertent, painless scalp mass that grew slowly. There were no significant differences between males and females in these cases (53.7% vs. 46.3%). The location of the lesions was random, and 11 cases occurred on the anterior fontanelle.

**Fig. 1:**
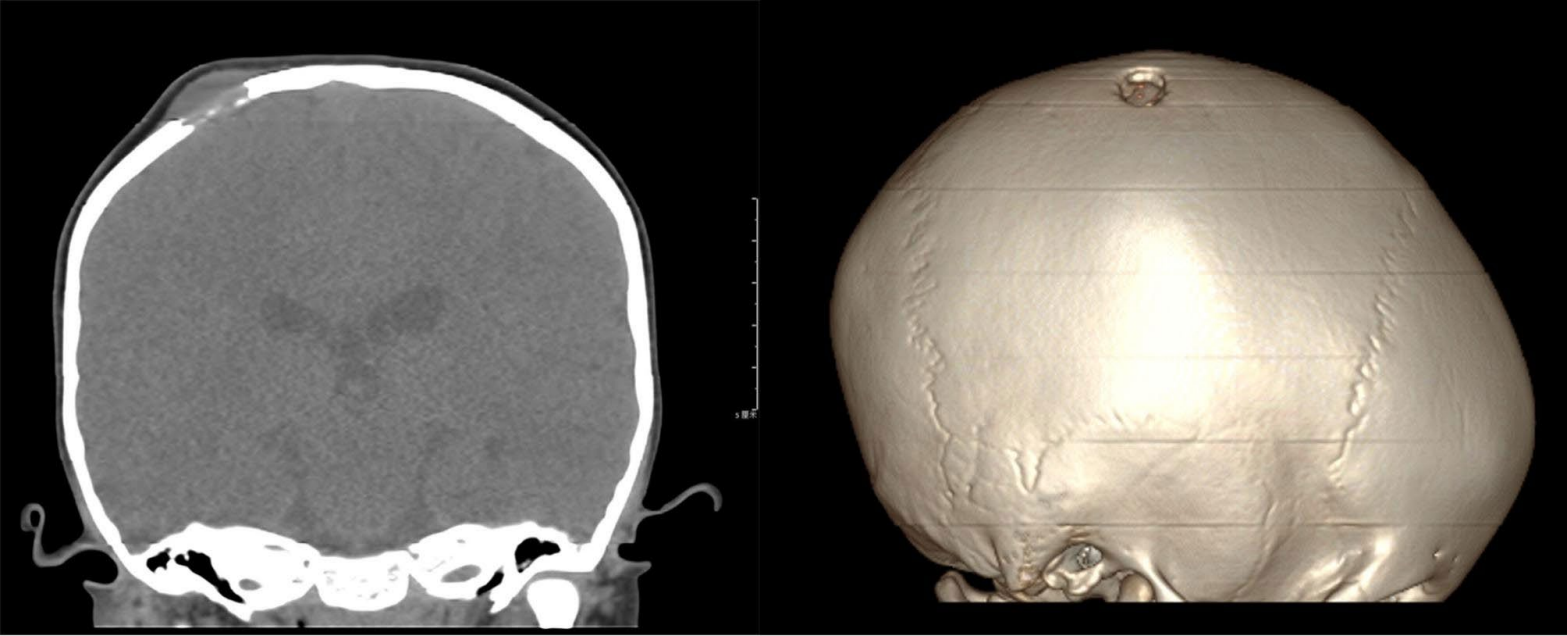
The LCH CT with cranial lesion, the CT showed bony destruction of the skull with chisel-like changes with ill-defined borders

**Fig. 2:**
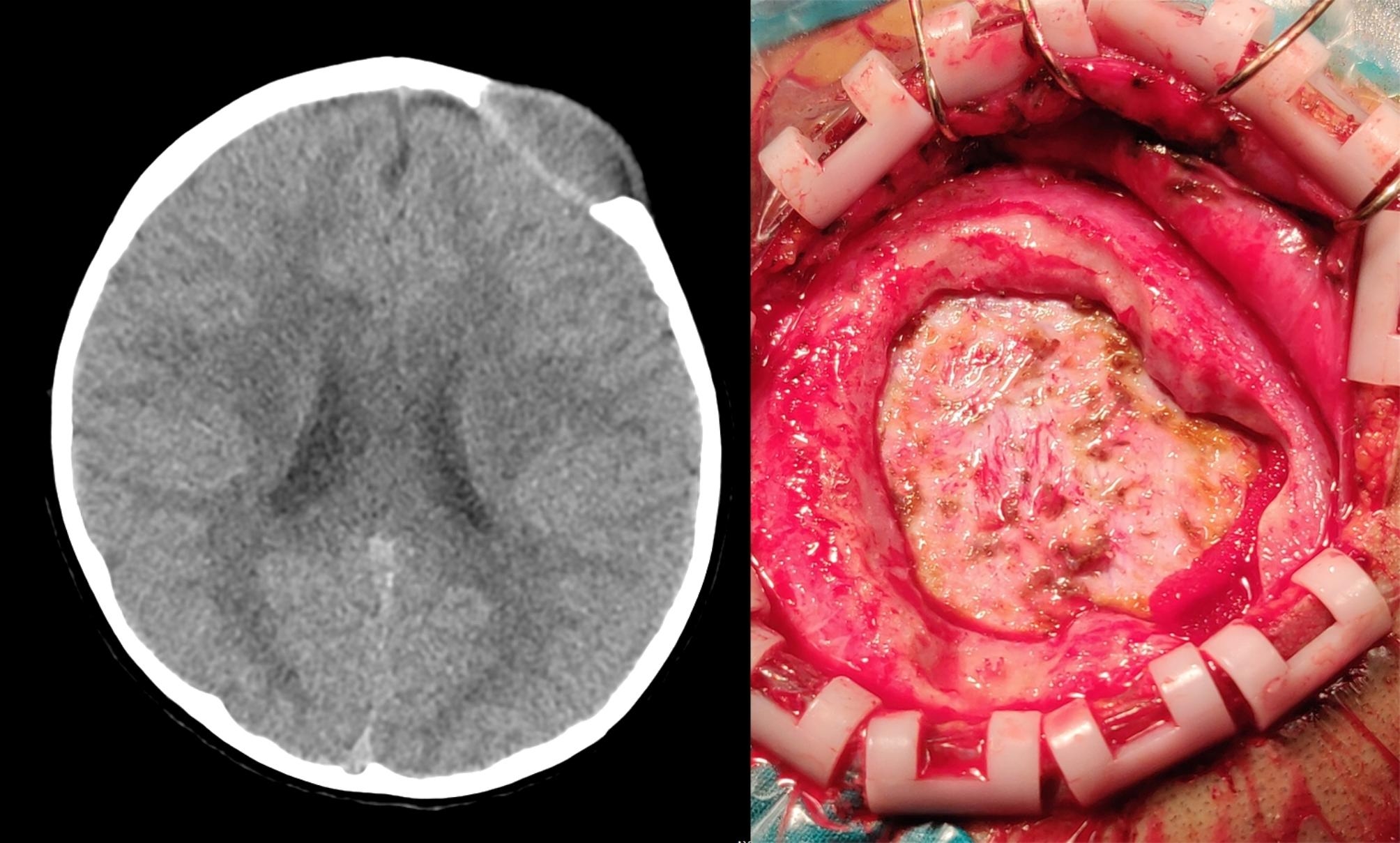
The Rosai-Dorfman disease CT and surgical picture, the CT (left) showed the fusiform soft tissue lesions are located in frontal subcutaneously continue to the skull, forming a large 5*5 cm defect on skull, and the surgery picture (right) showed a large skull defect was revealed after the mass was removed

A total of 23 cases were diagnosed with LCH after resection of the lesions. Nearly half of 13/23 LCH patients were multiple parts involved in the body, and 10/23 LCH patients were single part involved, which could further evaluation of the condition. Subsequent chemotherapy proceeded with standard recession, and all patients alive; 1 case developed a Wilms tumor at the age of 1 year old, with right kidney and tumor resection. The tumor recurred at 3 years of age at the pelvic para-ovaries, and tumor resection was performed. The skull lesion occurred at age of 4 years old, with surgical resection and histologically confirmed LCH. The CT of this patient showed bony destruction of the skull with chisel-like changes and ill-defined borders (Fig. 1). One case was diagnosed with Rosai–Dorfman disease, where CT imaging showed that fusiform soft tissue lesions located in the frontal subcutaneously continue to the skull, forming a large 5*5 cm defect on the skull (Fig. 2). The surgery image showed a large skull defect after the mass had been removed (Fig. 2).

Overall, 6 cases of vascular malformation and 4 cases of hemangioma were ascertained with vascular-related lesions. The pathology diagnosis of 6 vascular malformation was Arteriovenous malformations (AVMs), which absence of intervening capillaries between arteries and veins, presenting with pulsatile scalp mass. Pre-surgery ultrasound diagnosed abundant blood flow signals within the lesions in scalp AVMs cases.

A total of 27 children were diagnosed with meningocele, with a male-to-female ratio of 2:1, where 24 patients were within 1 month of age. All lesions were located at or near the midline of the skull, 9 of which were located at the top, and 18 were situated in the occipital region; none of the patients showed signs of hydrocephalus on imaging.

**Fig. 3:**
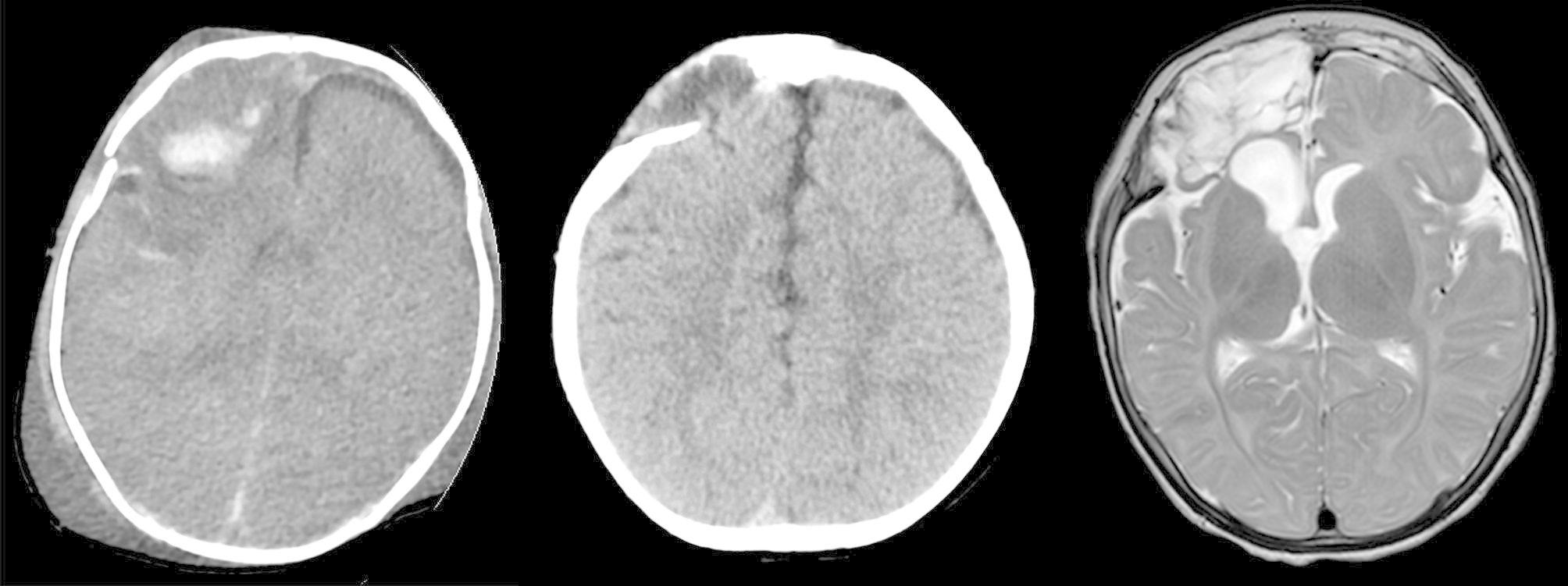
Growing skull fracture, CT and MRI scan. Left image, linear fracture immediately posttrauma, right frontal lobe. Middle and right image, 7 months later, note the herniation of the skull defect and degenerated brain tissue

Growth fractures were diagnosed for 7 patients (Fig. 3), all of whom suffered head trauma below the age of 1 year old. All patients got linear fracture at the trauma, gradually unhealed and grown(4 on the forehead and 3 on the top of the skull). The special case of growth fractures was 1-month-old infant was admitted to the emergency department of the authors’ hospital due to severe craniocerebral trauma resulting from a car accident and was discharged following conservative treatment. The child developed a progressively enlarged mass on the right side of the forehead accompanied by pulsation; the size of the skull defect was 3*1.5 cm. Epilepsy and mental retardation were observed in the children during follow-up.

A total of 12 patients were diagnosed with myofibroma after lesion resection, and cranial infiltration could be observed on CT in some cases. Fluorescence in situ hybridization results in one of the patients revealed that the USP6 gene had been rearranged by the t(17;22)(p13;q12.3)MYH9-USP6 isochromosomal translocation. One patient was diagnosed with infantile fibroids; another patient was diagnosed with myofibroma accompanied by a neck mass, as well as multiple bone lesions on the right parietal bone, temporal bone, bilateral mastoid, and left orbit. The neck mass was surgically removed, and no tumor progression was observed during follow up.

**Fig. 4:**
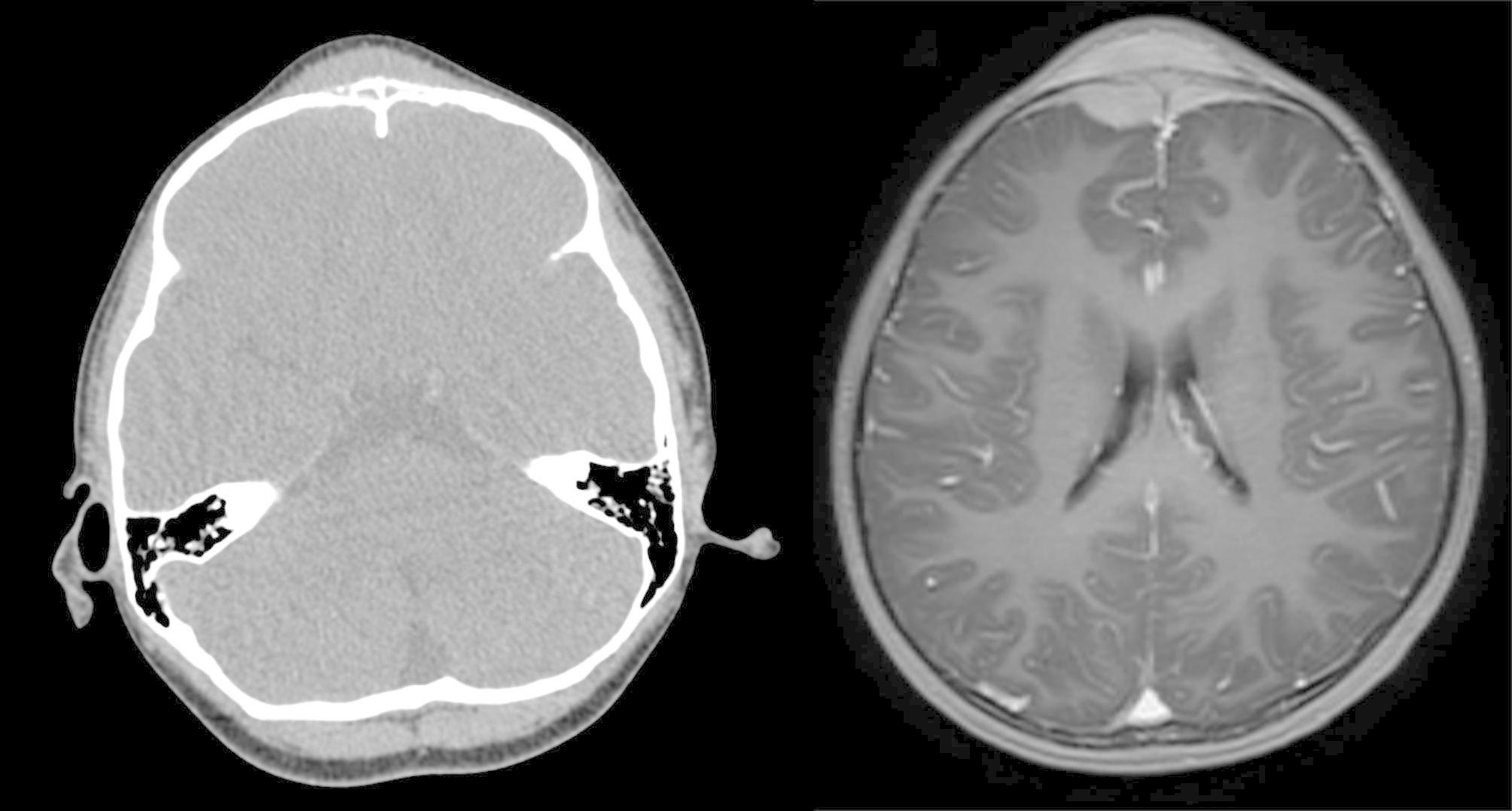
B lymphoblastic lymphoma of the bone, CT and MRI scan. Left image, bone destruction of frontal bone with soft tissue mass. Right image, frontal mass, fusiform mass on inner and outer plates of skull, accompanied by compression of brain tissue

For 5 patients, lesions were pathologically confirmed to be malignant post-surgery. This included 2 cases of hemangioendothelioma, one of which was diagnosed as epithelioid hemangioendothelioma, and this patient underwent reoperation due to tumor recurrence at the primary surgical site. One case of neuroblastoma with a progressively enlarged mass on the forehead was treated surgically, and the postoperative pathological diagnosis was neuroblastoma (poorly differentiated type). Further examination showed that the primary tumor was located in the adrenal gland, with multiple lymph node metastases throughout the body. One case of B-lymphoblastic lymphoma presented with a progressively enlarged mass on the forehead. A CT scan showed multiple bone destructions in the skull and scapula (Fig. 4). A tumor on the forehead with a size of 9*7 cm was found to be located under the periosteum during surgery, invading the skull and dura mater; primary B-lymphoblastic lymphoma of the bone was diagnosed. In addition, 1 case was diagnosed as being a myeloid sarcoma. Overall, the malignancy have a relatively poor prognosis, two patients got tumor recurrence.

## Discussion

In the present paper, the authors summarized children’s scalp and skull lesions recorded in a regional medical center’s department of neurosurgery. Differential diagnoses of the lesions in children should consider both common and rare lesions with a variety of symptoms and imagological examinations. The study elaborated on several lesions diagnosed in children, including clinical information and histological types, to which clinicians should attach significant importance.

Dermoid cysts are benign congenital lesions that typically appear on the surface of the skull and originate from entrapment of the surface ectoderm from the lines of embryonic fusion [3, 4]. The Dermoid cysts were most frequently represented before life, and then gradually enlarge accompany with or without extension into surrounding structures [5], indicating persistence and continued enlargement. Existing studies showed that approximately a quarter of dermoid cysts were located on the anterior fontanel [6, 7]. The data of the present study indicated the most common site of dermoid cysts to be occipital of skull(34/108 = 31.5%). The early surgical resection of dermoid cysts presents an excellent prognosis [8, 9]. Most lesions are easily removed completely, but recurrences may occur if complete removal is not achieved.

Langerhans cell histiocytosis is a rare histiocytic disorder most commonly characterized by solitary or multiple osteolytic bone lesions showing bean-shaped nuclei on biopsy and histiocyte infiltration. Skeletal involvement was present at diagnosis in 77% of patients [10] with the skull most commonly affected in children (40%) [11]. Some cranial lesions are not only osteolytic but may also be accompanied by a mass that invades the dura, leading to an increased level of disease risk assessment. The diagnosis of LCH is based on biopsy, and since LCH bone lesions can be completely/nearly recovered with curettage or one-off chemotherapy, surgeons should not perform extensive excision in these cases. Solitary LCH lesions of the skull show a low incidence of recurrence (0-1.5%), but this may be as high as 31% in other reports [12, 13].

The vascular malformation of AVMs in the scalp, also known as cirsoid aneurysms, are defined according to the absence of intervening capillaries that connect between superficial arteries and draining veins, presenting with a pulsatile, enlarging mass that may be associated with headaches, tinnitus, and bleeding [14]. Children’s scalp AVMs can have a changeable neoplasm size, based on where they occur on the body; these may increase in the supine position but decrease in an upright position. Computed tomography angiography, and B-scan ultrasonography examination showed abundant blood flow signals in the presence of AVMs, some of blood flow signals could extend into the sigmoid sinus [15]. Because curettage as a mode of therapy has been reported to entail both increased bleeding and chance of recurrence, en bloc resection of the normal margin has been shown to be an effective treatment option with less chances of recurrence [16, 17].

Meningoceles are herniations of the meninges and cerebrospinal fluid and are characterized according to the type of intracranial content protruding from the skull opening [18]. Bui et al. [19] reported hydrocephalus in 60% of occipital encephalocele cases, while epilepsy was confirmed in 17% of such cases. All of the patients included in the current study were free of brain bulges and had small skull defects. Surgical procedures only required ligation and resection of the root of the bulge and, accordingly, the prognosis was generally positive. However, for cases of suspected meningoencephalocele, puncture, biopsy, or blind excision should not be performed until the source of the tumor has been confirmed by an imaging examination.

Growing skull fractures occur mainly in infants and young children (younger than 3 years) and manifest as a persistent cystic or pulsatile mass and progressive widening of skull fracture sutures after trauma. Once diagnosed, surgery should be performed as soon as possible to avoid persistent brain damage and neurological complications. Singh I et al. [20] studied 905 children with skull fractures younger than 5 years of age, 6 of whom had growth fractures, all of which developed within 2 years following birth. Comminuted fracture patterns and greater diastasis width increased the likelihood of growth fractures occurring [21]. Cephalhematomas usually become calcified, but it has been suggested that subgaleal hematomas do not calcify [22, 23].

Myofibromas are rare tumors that typically occur in infants and young children [24]. Oudijk et al. [25] reported on 114 patients for whom these tumors occurred in 65% before the age of 2 years. In our study, there were 5 cases (5/12, 41.7%) of children below the age of 1 year, and 9 cases (9/12, 75%) younger than 6 years old with this type of tumor, which was consistent with the literature reports [25]. Tumors are most commonly found in the head and neck, followed by the trunk, the skin, or the subcutaneous tissue of the upper and lower extremities. The main clinical manifestation is a painless solitary mass, which can comprise not only soft tissue but also bone and internal organs. Although complete resection is the surgical treatment of choice for fibroids, positive surgical margins do not adversely affect outcomes. Mahajan et al. [26] reported that 26 patients had incomplete tumor resection, but none evidenced tumor progression. Childhood myofibromas demonstrated clinical variability and could spontaneously regress; in these cases, observation after conservative excision or biopsy may be sufficient for some patients. In the current study, myofibromatosis manifested as a cranial soft tissue mass with surrounding bone destruction (according to CT scans), and it was necessary to differentiate it from cholesteatoma, LCH, and metastases using histopathological findings. Ubiquitin-specific peptidase 6 (USP6) is a deubiquitinating enzyme gene on chromosome 17p13 with functions of inflammatory signaling, cell transformation and protein turnover. USP6 rearrangements was associated with myofibroblastic tumors, which could be further biomarker in myofibromas.

Malignant tumors of the scalp in children were less common, with a total of 5 cases (5/228, 2.19%) in the current study cohort. However, malignancies can often progress rapidly, with imaging characteristics evidencing a poorly defined lytic lesion with an aggressive appearance, extension into bordering soft tissue, and intense heterogeneous enhancement [27]. In addition, for malignant tumors with scalp tumors as the first manifestation, a systemic examination should be performed to determine whether lesions are present elsewhere. Surgical resection should be performed as soon as possible if scalp tumors are suspected of being malignant. Subsequent treatment should be carried out after the pathological results have been confirmed.

A limitation of the present research is that the follow-up period was relatively short, and further prognosis and some malignant recurrences could not be observed. Furthermore, the medical center’s clinical data comprised with more complicated and rare disease, which could not fully reflect the exact population information of pediatric scalp neoplasms. Thus, additional studies comprising long-term follow up are needed for better evidence in the field of pediatric scalp and skull lesions and their treatment.

## Conclusion

The incidence of children’s scalp and skull lesions varied from being common and benign to uncommon and/or malignant. These lesions should be diagnosed early using both clinical symptoms and imaging information. Furthermore, patients can benefit from early surgical resection. Significant importance should be attached to malignant/ special case lesions.

## Data Availability

All data generated or analysed during this study are included in this article. Further enquiries can be directed to the corresponding author.

## Abbreviations

AVMs: Arteriovenous malformations
CSF: Cerebrospinal Fluid
CT: Computed Tomography
FISH: Fluorescence in situ hybridization
GSF: Growing skull fracture
LCH: Langerhans cell histiocytosis

## References

[1] Prodinger CM, Koller J, Laimer M (2018). Scalp tumors. J Dtsch Dermatol Ges.

[2] Morón FE, Morriss MC, Jones JJ, Hunter JV. Lumps and bumps on the head in children: use of CT and MR imaging in solving the clinical diagnostic dilemma. Radiographics. 2004 Nov-Dec;24(6):1655-74. 10.1148/rg.246045034. PMID: 15537975. 10.1148/rg.246045034 15537975

[3] Ponce-Ayala A, Navarro-Garcia de Llano JP, Degollado-Garcia J, Hernández-Álvarez N, Mendizabal-Guerra R (2021). Anterior Fontanelle Dermoid Cyst: Surgical technique. Cureus.

[4] Khalid S, Ruge J (2017). Considerations in the management of congenital cranial dermoid cysts. J Neurosurg Pediatr.

[5] Orozco-Covarrubias L, Lara-Carpio R, Saez-De-Ocariz M, Duran-McKinster C, Palacios-Lopez C, Ruiz-Maldonado R. Dermoid cysts: a report of 75 pediatric patients. Pediatr Dermatol. 2013 Nov-Dec;30(6):706–11. 10.1111/pde.12080. Epub 2013 Mar 14. PMID: 23488469. 10.1111/pde.12080 23488469

[6] Pannell BW, Hendrick EB, Hoffman HJ, Humphreys RP (1982). Dermoid cysts of the anterior fontanelle. Neurosurgery.

[7] Reissis D, Pfaff MJ, Patel A, Steinbacher DM (2014). Craniofacial dermoid cysts: histological analysis and inter-site comparison. Yale J Biol Med.

[8] Udina C, Calligaris L, Berti I, Cattaruzzi E, Barbi E (2020). Inclusion cyst of anterior fontanelle. Arch Dis Child.

[9] Prior A, Anania P, Pacetti M, Secci F, Ravegnani M, Pavanello M, Piatelli G, Cama A, Consales A (2018). Dermoid and epidermoid cysts of scalp: Case Series of 234 consecutive patients. World Neurosurg.

[10] Grois N, Pötschger U, Prosch H (2006). Risk factors for diabetes insipidus in langerhans cell histiocytosis. Pediatr Blood Cancer.

[11] Slater JM, Swarm OJ (1980). Eosinophilic granuloma of bone. Med Pediatr Oncol.

[12] Martinez-Lage JF, Capel A, Costa TR (1992). The child with a mass on its head: diagnostic and surgical strategies. Child’s Nerv Syst.

[13] Rawlings CE, Wilkins RH (1984). Solitary eosinophilic granuloma of the skull. Neurosurgery.

[14] Shenoy SN, Raja A (2004). Scalp arteriovenous malformations. Neurol India.

[15] Kanth AM, Ricci JA, Adetayo OA (2019). Diagnosis and treatment of scalp arteriovenous malformations with intracranial extension. J Craniofac Surg.

[16] Claus EB, Persing JA, Winn HR (2004). Scalp tumors. Youmans neurological surgery.

[17] Fewel WE, Gebarski SS, Hoff JT, Winn HR (2004). Skull tumors. Youmans neurological surgery.

[18] Markovic I, Bosnjakovic P, Milenkovic Z (2020). Occipital encephalocele: cause, incidence, neuroimaging and Surgical Management. Curr Pediatr Rev.

[19] Bui CJ, Tubbs RS, Shannon CN (2007). Institutional experience with cranial vault encephaloceles. J Neurosurg.

[20] Singh I, Rohilla S, Siddiqui SA, Kumar P (2016). Growing skull fractures: guidelines for early diagnosis and surgical management. Childs Nerv Syst.

[21] Lopez J, Chen J, Purvis T (2020). Pediatric Skull fracture characteristics Associated with the development of Leptomeningeal Cysts in Young Children after Trauma: a single Institution’s experience. Plast Reconstr Surg.

[22] Cummings TJ, George TM, Fuchs HE (2004). The pathology of extracranial scalp and skull masses in young children. Clin Neuropathol.

[23] Willatt JM, Quaghebeur G (2004). Calvarial masses of infants and children. A radiological approach. Clin Radiol.

[24] Wiswell TE, Davis J, Cunningham BE, Solenberger R, Thomas PJ (1988). Infantile myofibromatosis: the most common fibrous tumor of infancy. J Pediatr Surg.

[25] Oudijk L, den Bakker MA, Hop WC (2012). Solitary, multifocal and generalized myofibromas: clinicopathological and immunohistochemical features of 114 cases. Histopathology.

[26] Mahajan P, Hicks J, Chintagumpala M, Venkatramani R (2017). Myofibroma in Infancy and Childhood. J Pediatr Hematol Oncol.

[27] Choudhary G, Udayasankar U, Saade C, Winegar B, Maroun G, Chokr J (2019). A systematic approach in the diagnosis of paediatric skull lesions: what radiologists need to know. Pol J Radiol.

